# *DSM-5* Changes, COVID-19, and ADHD Diagnosis Rates in Individuals Younger Than 30 Years

**DOI:** 10.1001/jamanetworkopen.2026.5775

**Published:** 2026-04-08

**Authors:** Zishan Cui, Anshula Ambasta, Wade Thompson, Ken Bassett, Greg Carney, Colin Dormuth

**Affiliations:** 1 Department of Anesthesiology, Pharmacology & Therapeutics, University of British Columbia, Vancouver, Canada; 2 Department of Medicine, University of British Columbia, Vancouver, Canada

## Abstract

This cohort study of youth in British Columbia examines changes in attention-deficit/hyperactivity disorder diagnosis rates following changes in *Diagnostic and Statistical Manual of Mental Disorders* (Fifth Edition) and the COVID-19 pandemic.

## Introduction

Attention-deficit/hyperactivity disorder (ADHD) is a common neurodevelopmental disorder among children and adolescents.^1^ It remains unclear how recent increases in ADHD diagnoses relate to changes introduced in *Diagnostic and Statistical Manual of Mental Disorders* (Fifth Edition) (*DSM-5*),^2^ or whether the COVID-19 pandemic contributed to short-time surges or longer-term shifts.^3^ Most ADHD studies have focused on prevalence rather than incidence, which may obscure emerging patterns.^4^ Using 2 decades of data from British Columbia, Canada, this study examined whether ADHD diagnosis rates changed over time in association with *DSM-5* implementation and the COVID-19 pandemic.

## Methods

We used British Columbia linked administrative data capturing physician visits, hospitalizations, prescription dispensations, and demographic information (eMethods in Supplement 1). We constructed a retrospective cohort of residents aged 3 to 29 years who were enrolled in British Columbia’s universal health plan from 2003 to 2023. Analyses were stratified by sex and age group: preschool (ages 3 to 5 years), elementary (6 to 12 years), high school (13 to 17 years), and young adult (18 to 29 years). This study was approved by the University of British Columbia research ethics board with informed consent waived because data were deidentified. Our study followed the Strengthening the Reporting of Observational Studies in Epidemiology (STROBE) reporting guideline for cohort studies.

ADHD diagnoses were identified using a validated algorithm based on physician visits (*International Classification of Diseases, Ninth Revision *[*ICD-9*] code 314), hospitalizations (*International Statistical Classification of Diseases and Related Health Problems, Tenth Revision *[*ICD-10*] code F90), or ADHD-specific prescriptions.^5^ To ensure incident diagnoses, we required at least 3 years of provincial health plan enrollment and excluded individuals with prior ADHD diagnosis. We calculated annual incidence by age-sex stratum and employed interrupted time-series analyses using generalized least squares regression to estimate level (intercept) and trend (slope) changes associated with the *DSM-5* and COVID-19 interventions, excluding 2013 (*DSM-5* transition) and 2020 (pandemic-related care disruptions). Analyses were conducted using SAS Enterprise Guide version 7.1 (SAS Institute Inc).

## Results

A total of 2 743 914 children and young adults aged 3 to 29 years were included, of whom 1 376 580 (50.8%) were male. Between 2003 and 2023, 110 874 males (805.4 per 10 000; 95% CI, 800.7-810.2 per 10 000) and 74 264 females (543.1 per 10 000; 95% CI, 539.3-547.0 per 10 000) were newly diagnosed with ADHD (Table). There was increasing incidence of ADHD diagnoses in the cohort prior to intervention (2003-2012) (Figure). Following *DSM-5* implementation (2014-2019), incidence accelerated among elementary school–aged male children (annual change, 9.8 increase per 10 000; 95% CI, 7.2-12.3 per 10 000) and female children (3.5 increase per 10 000; 95% CI, 1.0-6.0 per 10 000). High school–aged male adolescents (7.8 increase per 10 000; 95% CI 5.8-9.9 per 10 000) and female adolescents (6.7 increase per 10 000; 95% CI, 4.6-8.7 per 10 000) showed nearly parallel increases. Post–COVID-19 (2021-2023), the largest immediate incidence increases were observed among high school–aged female adolescents, from 86.8 per 10 000 (95% CI, 81.4-92.6 per 10 000) in 2019 to 203.7 per 10 000 (95% CI, 195.5-212.4 per 10 000) in 2021, and among female young adults, from 54.1 per 10 000 (95% CI, 51.4-56.8 per 10 000) to 183.2 per 10 000 (95% CI, 178.3-188.2 per 10 000). Meanwhile, the most pronounced trend accelerations post–COVID-19 were observed among elementary school–aged male children (annual change, 23.7 increase per 10 000; 95% CI, 16.5-30.8 per 10 000) and female changed (20.2 increase per 10 000; 95% CI, 13.0-27.3 per 10 000).

**Table.** Incidence of Attention-Deficit/Hyperactivity Disorder (ADHD) Diagnoses Stratified by Age Group and Sex, 2003-2023

Group	Individuals, No.^a^	Incidence cases^b^	Incidence, No./10 000 (95% CI)
Overall			
Male	1 376 580	110 874	805.4 (800.7-810.2)
Female	1 367 334	74 264	543.1 (539.3-547.0)
Age 3-5 y			
Male	564 268	9436	167.2 (163.9-170.6)
Female	534 721	2683	50.2 (48.3-52.1)
Age 6-12 y			
Male	657 317	58 188	885.2 (878.0-892.5)
Female	635 402	23 665	372.4 (367.7-377.2)
Age 13-17 y			
Male	588 048	17 429	296.4 (292.0-300.8)
Female	596 574	16 026	268.6 (264.5-272.8)
Age 18-29 y			
Male	902 740	25 821	286.0 (282.6-289.5)
Female	934 819	31 890	341.1 (337.4-344.9)

^a^ Residents enrolled in British Columbia’s provincial medical service plan for at least 3 years, or from age 6 or younger, who had never been previously diagnosed with ADHD.

^b^ British Columbian residents meeting the predefined ADHD case definition.

**Figure.**
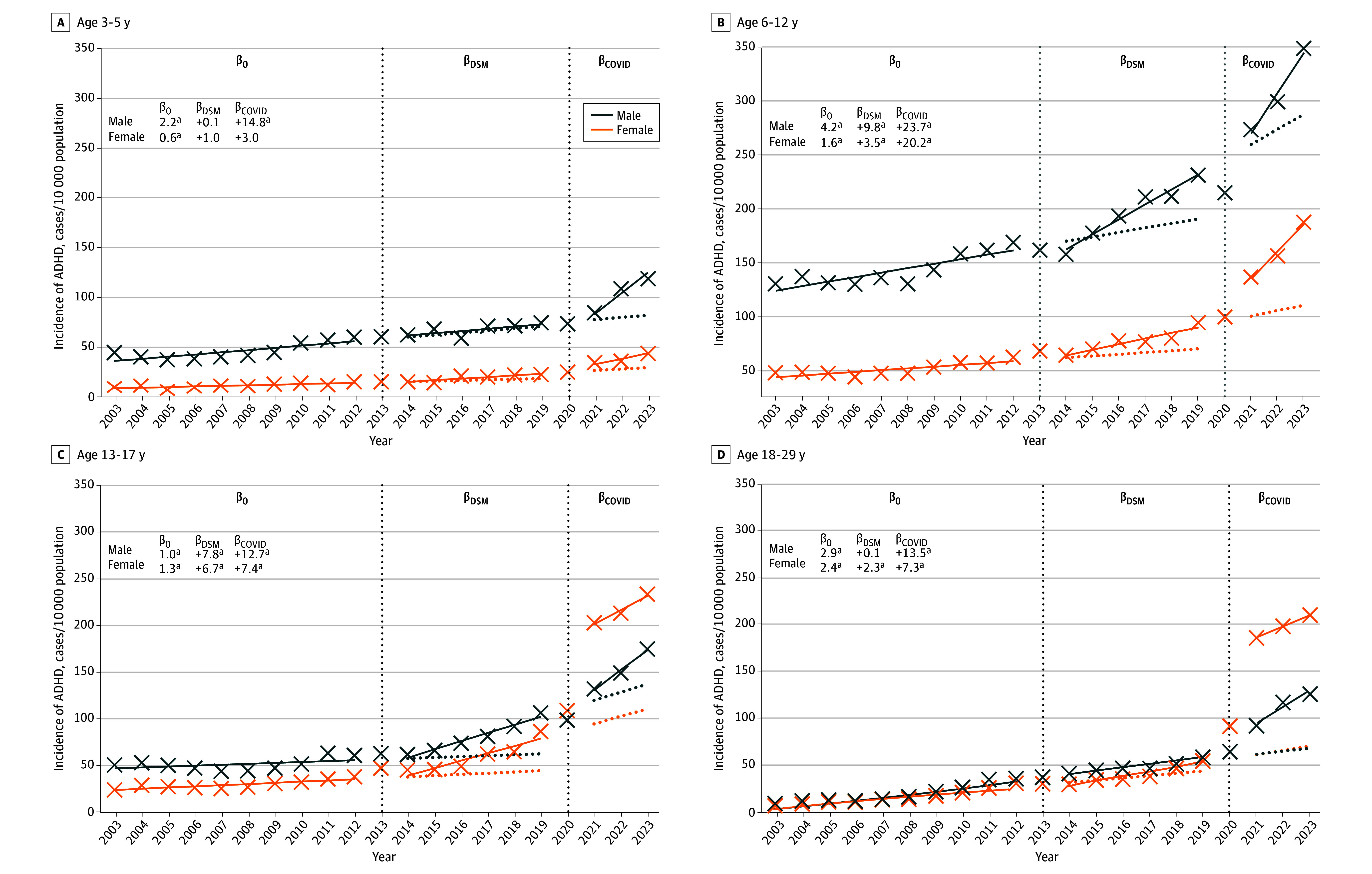
Line Graph of Annual Incidence of Attention-Deficit/Hyperactivity Disorder (ADHD) Diagnoses in British Columbia, 2003-2023 Estimates were baseline trends and trend changes from interrupted time-series (ITS) models: β_0_ indicates the baseline trend (2003-2012); β_DSM_, the additional annual trend changes following *Diagnostic and Statistical Manual of Mental Disorders* (Fifth Edition) (*DSM-5*) implementation (2014-2019), relative to the baseline trend; β_COVID_, the additional annual trend changes following the COVID-19 pandemic (2021-2023) relative to the prepandemic trend (*β_0_ + β_DSM_*). ^a^*P* < .05 for statistically significant trend-change estimates.

## Discussion

Incidence of ADHD diagnoses increased over 2 decades in British Columbia, and appeared to accelerate following the implementation of *DSM-5* changes and the COVID-19 pandemic. The sharpest postpandemic increases occurred among female adolescents and young adults, surpassing same-aged males for the first time on record and mirroring a similar but more transient pattern reported in Finland.^6^ Increasing public and clinical awareness of ADHD may have contributed to greater recognition and help-seeking. Revisions in *DSM-5*, including changes to diagnostic wording emphasizing functional interference rather than clinically significant impairment, may have broadened diagnostic identification.^2^ Pandemic-related stressors may have further intensified ADHD symptoms and facilitated the identification of previously unmet needs, with female adolescents and young adults potentially being more vulnerable to social disruptions than males.^3^

Limitations of this study include under-capture of individuals with ADHD who were undiagnosed or managed exclusively through private settings and the inability to infer causality between *DSM-5* implementation, the pandemic, and diagnostic patterns. Nonetheless, the sustained postpandemic incidence increase, especially among females, highlights the importance of monitoring age- and sex-specific diagnostic trajectories and ensuring appropriate ADHD assessment and service planning.

## Notes

Supplement 1. eMethods.

Supplement 2. Data Sharing Statement
