# Supplementary material for: DSM-5 Changes, COVID-19, and ADHD Diagnosis Rates in Individuals Younger Than 30 Years
Source: JAMA Netw Open. 2026 Apr 8;9(4):e265775. doi: 10.1001/jamanetworkopen.2026.5775 (PMC13063071; doi:10.1001/jamanetworkopen.2026.5775)
Supplement: Supplement 1. — eMethods. [file jamanetwopen-e265775-s001.pdf]

Supplemental Online Content

Cui Z, Ambasta A, Thompson W, Bassett K, Carney G, Dormuth C. *DSM-5* changes, COVID-19, and ADHD diagnosis rates in individuals younger than 30 years. *JAMA Netw Open.* 2026;9(4):e265775. doi:10.1001/jamanetworkopen.2026.5775

eMethods.

This supplemental material has been provided by the authors to give readers additional information about their work.

© 2026 Cui Z et al. *JAMA Network Open*.

Supplement – eMethods

Data sources

This study used linked data from the British Columbia provincial healthcare administrative data warehouse, which captures physician services (Medical Services Plan), prescription medications dispensed from community pharmacies (PharmaNet), hospital admissions (Discharge Abstract Database), and emergency hospital visits (National Ambulatory Care Reporting System).[1] Demographic and mortality data were extracted from the Ministry of Health Client Roster, which includes records of individuals who have lived in BC since 1986. The UBC Research Ethics Board approved this study with a waiver of informed consent because deidentified administrative data were used.

Study Design

We constructed a retrospective cohort of male and female BC residents aged 3 to 29 years who were registered in the provincial universal health insurance program between January 1, 2003, and December 31, 2023. The provincial health plan in BC provides universal, publicly funded coverage for medically necessary services to all residents, regardless of income. For each calendar year (x), age was calculated using x minus the year of birth, without accounting for the exact date or month of birth. This approach was used to reflect the December 31 annual cut-off date for school enrollment in BC.

Statistical Analyses

Based on a previously validated ADHD diagnosis definition (sensitivity: 83.2%; specificity: 98.6%),[2] an ADHD diagnosis was defined as meeting any of the following criteria: (1) one filled prescription for an ADHD-specific medication, including methylphenidate, lisdexamfetamine,

© 2026 Cui Z et al. *JAMA Network Open*.

dextroamphetamine, mixed amphetamine salts, atomoxetine, or guanfacine;[3] (2) one hospitalization with an ICD-10 code F90; or (3) one physician visit with an ICD-9 code 314, accompanied within one year by either another physician visit with the same diagnosis code, filled prescription for an ADHD-specific drug, or a hospitalization for ADHD. The incidence numerator included the first recorded ADHD diagnosis for each individual. The denominator included all BC residents who had no prior ADHD diagnosis since the year 2000. To ensure accurate identification of incident cases, both the numerator and denominator were restricted to individuals who either resided in BC before age six, or had been continuously enrolled in the provincial health plan for at least three years. Gaps in provincial health plan coverage up to 28 days were ignored and considered uninterrupted residency.

Next, to determine whether the trends observed in incident ADHD diagnoses were associated with DSM-5 diagnostic criteria updates in 2013 and the COVID-19 pandemic, we conducted interrupted time-series (ITS) analyses to quantify the impact of these events on incident ADHD diagnoses. This design allowed us to examine the immediate change (change in intercept) and change over time (change in slope) in incident ADHD diagnoses while controlling for pre-existing trends.[4] For each age group, we fitted a generalized least squares linear regression model to yearly incidence proportions across three key periods: the DSM-IV-TR era (2003–2012), the DSM-5 implementation period (2014–2019), and the post-COVID era (2021–2023). Each model included an intercept representing baseline incidence at the start of the study period in 2003, along with the pre-2013 trend corresponding to the DSM-IV-TR era. To evaluate the impact of the 2013 diagnostic criteria update, each model included an intercept change variable and a slope variable post-2013. We excluded data from year 2013 to account for the potential transitional effects during the implementation of the new diagnostic criteria. Similarly, to assess the impact of the COVID-

© 2026 Cui Z et al. *JAMA Network Open*.

19 pandemic, each model included an immediate level-change variable and a trend-change variable post-COVID. Data from 2020 were also excluded to address a potential temporary drop in diagnoses due to limited access to care during the pandemic in BC.[5] Data were extracted using PLSQL Developer, and statistical models were fitted using SAS Enterprise Guide 7.1. All statistical tests were two-sided, with a significance level set at 0.05.

Supplement References

1. Humphries KH, Rankin JM, Carere RG, Buller CE, Kiely FM, Spinelli JJ. Co-morbidity data in outcomes research: are clinical data derived from administrative databases a reliable alternative to chart review?. *J Clin Epidemiol*. 2000;53(4):343-349. doi:10.1016/s0895-4356(99)00188-2
2. Butt DA, Jaakkimainen L, Tu K. Prevalence and Incidence Trends of Attention Deficit/Hyperactivity Disorder in Children and Youth Aged 1-24 Years in Ontario, Canada: A Validation Study of Health Administrative Data Algorithms: Tendances de la prévalence et de l'incidence du trouble de déficit de l'attention/hyperactivité chez les enfants et les jeunes âgés de 1 à 24 ans, en Ontario, Canada: une étude de validation des algorithmes de données administratives de santé. Can J Psychiatry. 2024;69(5):326-336. doi:10.1177/07067437231213553
3. Clemow DB, Walker DJ. The potential for misuse and abuse of medications in ADHD: a review. *Postgrad Med*. 2014;126(5):64-81. doi:10.3810/pgm.2014.09.2801
4. Cruz M, Bender M, Ombao H. A robust interrupted time series model for analyzing complex health care intervention data. *Stat Med*. 2017;36(29):4660-4676. doi:10.1002/sim.7443
5. Chai Y, Man KKC, Luo H, et al. Incidence of mental health diagnoses during the COVID-19 pandemic: a multinational network study. *Epidemiol Psychiatr Sci*. 2024;33:e9. Published 2024 Mar 4. doi:10.1017/S2045796024000088

© 2026 Cui Z et al. *JAMA Network Open*.
