# Supplementary material for: DSM-5 Changes, COVID-19, and ADHD Diagnosis Rates in Individuals Younger Than 30 Years
Source: JAMA Netw Open. 2026 Apr 8;9(4):e265775. doi: 10.1001/jamanetworkopen.2026.5775 (PMC13063071; doi:10.1001/jamanetworkopen.2026.5775)
Supplement: Supplement 2. — Data Sharing Statement [file jamanetwopen-e265775-s002.pdf]

Data Sharing Statement

Cui. DSM-5 Changes, COVID-19, and ADHD Diagnosis Rates in Individuals Younger Than 30 Years. *JAMA Netw Open*. Published April 08, 2026. doi:10.1001/jamanetworkopen.2026.5775

Data
Data available: No

Additional Information
Explanation for why data not available: The data used in this study were obtained from population-based administrative health databases in British Columbia, Canada. These data contain personal health information and cannot be shared publicly due to provincial privacy legislation and data-sharing agreements. The analytic code and variable definitions used in this study are available upon request.
